# Targeting the N-cadherin/*β*-catenin axis with MSAB reverses malignant phenotypes in blast crisis of CML

**DOI:** 10.3389/fonc.2025.1657508

**Published:** 2025-10-16

**Authors:** Yingying Zhou, Songqin Jian, Lijun Lu, Xiaofang Li, Ruyi Qiu

**Affiliations:** ^1^ Department of Laboratory Medicine, Huangyan Hospital of Wenzhou Medical University, Taizhou First People’s Hospital, Taizhou, Zhejiang, China; ^2^ Hangzhou Mejia Biotechnology Co, Hangzhou, Zhejiang, China; ^3^ Department of Laboratory Medicine, Zhejiang Provincial Hospital of Traditional Chinese Medicine, Hangzhou, Zhejiang, China

**Keywords:** CML progression, exression profile, N-cadherin, β-catenin, MSAB, Wnt signaling

## Abstract

**Introduction:**

The molecular mechanisms underlying chronic myeloid leukemia (CML) progression from chronic phase (CP) to blast crisis (BC) remain incompletely understood. This study aimed to identify progression-specific genes and elucidate the role of N-cadherin (CDH2) in BC transformation.

**Methods:**

We analyzed the GSE4170 dataset to identify differentially expressed genes (DEGs) across CML phases. Functional annotations were performed via GSEA, GO, and KEGG analyses. The role of N-cadherin was validated using *in vitro* (KU812 cell line) and *in vivo* (nude mouse xenograft) models. The *β*-catenin inhibitor MSAB was employed for mechanistic studies.

**Results:**

Transcriptomic analysis identified 1,294 DEGs during CML progression, with 41 "progression-specific" genes showing consistent expression trends. Among these, N-cadherin was significantly upregulated in BC patient samples. Overexpression of N-cadherin in KU812 cells promoted cell cycle entry, accelerated tumor growth *in vivo*, and suppressed the expression of granulocyte surface differentiation antigens. MSAB treatment effectively reversed these malignant phenotypes. Furthermore, CDH2 mRNA was notably upregulated in advanced-phase samples compared to chronic-phase samples.

**Discussion:**

Our findings elucidate the role of CDH2 in CML progression through activation of the Wnt/*β*-catenin signaling pathway. Targeting the N-cadherin/*β*-catenin axis with MSAB may represent a novel therapeutic strategy for BC-CML.

## 1 Introduction

Chronic myeloid leukemia (CML) is driven by the BCR::ABL fusion tyrosine kinase and progresses gradually from a chronic phase of excessive mature myeloid cell proliferation to a fatal blast crisis (BC) resembling acute myeloid leukemia (AML), which is characterized by impaired differentiation. Although tyrosine kinase inhibitors (TKIs) can induce remission and prevent disease progression, a significant proportion of patients still advance to the blast phase with limited therapeutic options. The molecular mechanisms underlying this transformation are not fully elucidated. The leukemia stem cell (LSC) theory posits that each leukemia comprises various cell types, but only a small subset known as leukemia stem cells possesses the capability to sustain growth and perpetuate disease recurrence (1, 2). Using the NOD/SCID murine transplantation model (3), flow cytometry, and other experimental techniques, investigators have confirmed the presence of leukemia stem cells in AML, childhood T-lymphoblastic leukemia (4), and chronic myeloid leukemia (5). In these models, simultaneous interference with P53 and c-Myc, rather than BCR::ABL itself, synergistically induced apoptosis and differentiation of human leukemia stem cells (6). Recent studies indicate that progenitor cells can acquire self-renewal capacity through specific mutations, thereby generating malignant clones (3, 7). *BCR::ABL* alone fails to confer self-renewal capacity to GMPs; instead, aberrant activation of RUNX1/EVI1 (8), GATA-2 (9), and Msi2 (10) has been implicated in the acute transformation of CML. Experimental mouse models reveal that high-level expression of *BCR::ABL* alone is insufficient to induce leukemia, whereas co-expression with Setbp1, Hoxa10 or Hoxa9 produces aggressive leukemia within 3 weeks (11, 12). Moreover, disruption of CBX7 (13) markedly hindered proliferation and promoted differentiation of AML cell lines. Collectively, these data underscore the need to identify cooperating genetic events that drive CML progression. Nevertheless, the genetic events that propel CML from chronic to blast phase are not fully elucidated. Only a limited number of genetic abnormalities have been identified, such as isochromosome 17(p) causing TP53 disruption, and less commonly, deletion of the P15/P16 tumor suppressor genes (14, 15).

Most studies of N-cadherin have focused on solid tumors, with fewer on leukemia and even more limited emphasis on its involvement in the progression of CML. Studies on N-cadherin in hematopoiesis have likewise focused on normal hematopoietic stem and progenitor cells rather than leukemic counterparts. The objective of this study was to identify genetic alterations during the transition from chronic to blast phase, and to identify potential prognostic markers or therapeutic targets. Our bioinformatic re-analysis of the GSE4170 (16) dataset recapitulated the distinct molecular profiles of CML phases originally reported by Radich. However, with a focus on identifying progression-driving genes, we extended the analysis to identify a core set of genes exhibiting consistent expression trends across all three phases. We identified four candidate molecules that may contribute to CML progression. Through functional validation experiments, we confirmed that N-cadherin promotes proliferation and suppresses differentiation of Philadelphia chromosomes–positive myeloblast by affecting *β*-catenin signaling. We provide both *in vitro* and *in vivo* evidence supporting the role of N-cadherin in promoting proliferation and inhibiting differentiation of CML myeloblasts, along with elucidating the underlying mechanism.

## 2 Materials and methods

### 2.1 Data sources

GSE4170 (16), a published gene-expression dataset of chronic myeloid leukemia, together with corresponding raw CEL files, was downloaded from the GEO database. The dataset GSE4170 contains 119 samples from CML patients at various disease stages and follow-up time-points. To focus on untreated disease progression, we excluded post-treatment remission samples and retained 91 untreated cases: chronic phase (CP, n = 42), accelerated phase (AP, n = 17) and blast crisis (BC, n = 32). Raw CEL files (Affymetrix HG-U133 Plus 2.0) were background-corrected, quantile-normalized and log2-transformed using the RMA algorithm (affy package v1.74.0). Probe sets were annotated with NetAffx release 36. Differential expression analyses (limma v3.54.0) were performed for the following contrasts: CP vs AP, AP vs BC, and CP vs BC, using Benjamini–Hochberg FDR < 0.05 and |log2 fold change| ≥1 as significance thresholds. GSEA was carried out with clusterProfiler (v4.6.0, fgsea method); reported q-values are FDR-adjusted. We stated the significance threshold for GSEA, which is an FDR q-value < 0.25, as is standard and recommended for GSEA. A volcano plot illustrating differential genes among all three phases is provided as **Supplementary Figure S1**.

### 2.2 Patient samples

Peripheral-blood specimens from 7 chronic-phase and 7 accelerated- or blast-phase CML patients were obtained from Zhejiang Provincial Hospital of Traditional Chinese Medicine for validation of the microarray findings. The basic clinical characteristics of these patients are summarized in **Supplementary Table S1**.

### 2.3 Cell lines, culture, and transfection

The human CML cell line KU812 was provided by the National Collection of Authenticated Cell Cultures (Shanghai, China). Cells were maintained in RPMI-1640 medium (Sigma-Aldrich, St. Louis, USA) supplemented with 10% fetal bovine serum (Serana, Pessin, Brandenburg, Germany) and 1% (v/v) penicillin/streptomycin (Gibco, Thermo Fisher Scientific, Waltham, MA, USA) in an atmosphere of 5% CO2 at 37 °C. This cell line was STR-authenticated at the start of the experiment and tested for mycoplasma during the course of the study. The KU812 cell was infected with lentivirus for 72h to overexpress N-cadherin and then treated with 2 μg/mL puromycin for 72 hours to select stable cells. The multiplicity of infection (MOI) of the KU812 was 10. N-cadherin gene was cloned into the lentiviral expression vector EF-1a/GFP & Puro. EF-1a/GFP & Puro-SCRAMBLE was used as the negative control. Both of aforementioned lentiviruses were provided by Genepharma Technology (Shanghai, China).

### 2.4 Quantitative real-time PCR

Total RNA was extracted and purified using the RNAiso Plus kit (TaKaRa, Kusatsu, Japan). The extracted RNA was then reverse transcribed into cDNA using the PrimeScript™ RT Master Mix Kit (TaKaRa, Kusatsu, Japan). Subsequently, qPCR detection was performed using the TB Green^®^ Premix ExTaq™ (Tli RNaseH Plus) kit (TaKaRa, Kusatsu, Japan) on an ABI Prism^®^ 7500 real-time PCR detection system (Applied Biosystems, Thermo Fisher Scientific, Foster City, USA). The following gene-specific primer sequences were used: GSK-3*β*, forward, 5′-ACGGGACCCAAATGTCAAAC-3′, and reverse, 5′-ATAAGGATGGTAGCCAGAGG-3′; c-Myc, forward, 5′-TGCTGCCAAGAGGGTCAAG-3′, and reverse, 5′-GCGCTCCAAGACGTTGTGTGT-3′; CCND1, forward, 5′-GTCGCTGGAGCCCGTGAAA-3′, and reverse, 5′-CGGATGGATGTCGGTGTA-3′; CCNE1, forward, 5′-CACCTGACAAAGAAGATGATGACC-3′, and reverse, 5′-AAGAGGGTGTTGCTCAAGAAAGT-3′; CDK2, forward, 5′-CCAGGAGTTACTTCTATGCCTGA-3′, and reverse, 5′-TTCATCCAGGGGAGGTACAAC-3′; CDK4, forward, 5′-TCAGCACAGTTCGTGAGGTG-3′, and reverse, 5′-GTCCATCAGCCGGACAACAT-3′; CDK6, forward, 5′-TCTTCATTCACACCGAGTAGTGC-3′, and reverse, 5′-TGAGGTTAGAGCCATCTGGAAA-3′; N-cadherin, forward, 5′-CGGTGCCATCATTGCCATCCT-3’, and reverse, 5′-AGTCATAGTCCTGGTCTTCTTCTCCT-3’; *β*-catenin, forward, 5′-AAAGCGGCTGTTAGTCACTGG-3′, and reverse, 5′-GACTTGGGAGGTATCCACATCC-3′; GAPDH, forward, 5′-GGACCTGACCTGCCGTCTAG′, and reverse, 5′-TAGCCCAGGATGCCCTTGAG-3′. Relative expression of target genes were calculated by the 2^-ΔΔCt^ method, normalized to GAPDH.

### 2.5 Apoptosis assay, cell cycle assay

Apoptosis and cell cycle were assessed using a Annexin V/PI apoptosis kit (Share Bio, Shanghai, China) and a cell cycle staining kit (MultiSciences, Hangzhou, China) on a Flow Cytometer (DxFLEX, Beckman Coulter, Jiangsu, China) with a minimum flow rate that at least 10–000 events were acquired per sample, respectively.

### 2.6 Cell proliferation assay

The Cell Counting Kit-8 (Dojindo, Kumamoto, Japan) was utilized for proliferation analysis. Cells were plated in a 96-well culture plate. Subsequently, 10 μL CCK-8 reagent was added for 2 hours at 37 °C. The absorbance at 450 nm was then measured using a microplate reader. Cell viability measurements were presented as the relative optical density compared to the control group.

### 2.7 Flow cytometry

For expression of cell surface markers, cells were stained with fluorochrome-conjugated antibodies against CD34, CD117, CD33, CD13, CD11b, CD16, CD10 (Becton, Dickinson and Company, New Jersey, USA), and then analyzed by flow cytometry.

### 2.8 Formation of subcutaneous tumors in immunodeficient mice

Nude mice (SLAC ANIMAL, Shanghai, China) aged 4–5 weeks were pre-treated with cyclophosphamide for 3 days before cell inoculation. Cyclophosphamide was dissolved in saline to a concentration of 0.1g/ml and injected intraperitoneally at a dosage of 100mg/kg/day per nude mouse. The cells in logarithmic growth phase were collected, washed, and injected into the right axilla of nude mice at a number of 1×10^7^ cells per mouse. The mice were randomly divided into two groups based on the type of cells injected. The treatment group received inoculation with cells overexpressing N-cadherin, while the control group was inoculated with cells transfected with a control plasmid. Their body weight and tumor growth were monitored every other day. If a tumor formed, its size was measured with calipers.

### 2.9 Ethics statement

This study was approved by the Institutional Ethics Committee of Zhejiang University of Traditional Chinese Medicine (approval number: 2021- KL-099-01). The Ethics Committee has mandated that the size of solid tumors should not exceed 10% of the total weight of the animal, or alternatively, that the endpoint for observation should be when the tumor reaches a size of 3,000 - 3,500 mm^3^.

### 2.10 Western blot analysis

Methods of protein extraction and isolation were performed as described previously (17). Primary antibodies included: anti-CDH2, anti-GSK-3β, anti-β-catenin, anti-c-Myc, anti-CCNE1, anti-P53, and anti-Tubulin (Proteintech, Wuhan, China); anti-p-GSK-3β, anti-CCND1, anti-CDK2, and anti-CDK4 (Cell Signaling Technology, Danvers, Massachusetts, USA). Membranes were incubated with HRP-conjugated secondary antibodies (1:2500–1:5000; Jackson ImmunoResearch Laboratories, West Grove, PA, USA) for 1 h at RT after TBST washing. Protein bands were visualized using Western ECL Substrate (Bio-Rad, USA) and analyzed with ChemiDox XRS+ system.

### 2.11 Cytokine array

Equal numbers of cells were cultured for 48 h, and the resulting supernatants were analyzed by the magnetic bead method (CEGER, Jiangxi, China) on flow cytometry. A panel of 6 cytokines was analyzed: IL-2, IL-4, IL-6, IL-10, IFN-γ and TNF-α.

### 2.12 Statistical analysis

The experimental results were expressed as mean ± SD and analyzed by GraphPad Prism software (version 7.0; San Diego, CA, USA). Comparisons between two groups were analyzed using the unpaired two-tailed Student’s t-test. For multi-group comparisons, one-way or two-way analysis ANOVA with Tukey’s *post-hoc* test was applied. Multiple comparisons were corrected by Tukey’s or Bonferroni *post-hoc* tests as appropriate; adjusted p values are reported. A p-value of less than 0.05 was considered statistically significant.

## 3 Results

### 3.1 Identification of progression-specific genes in CML

**Figure 1:**
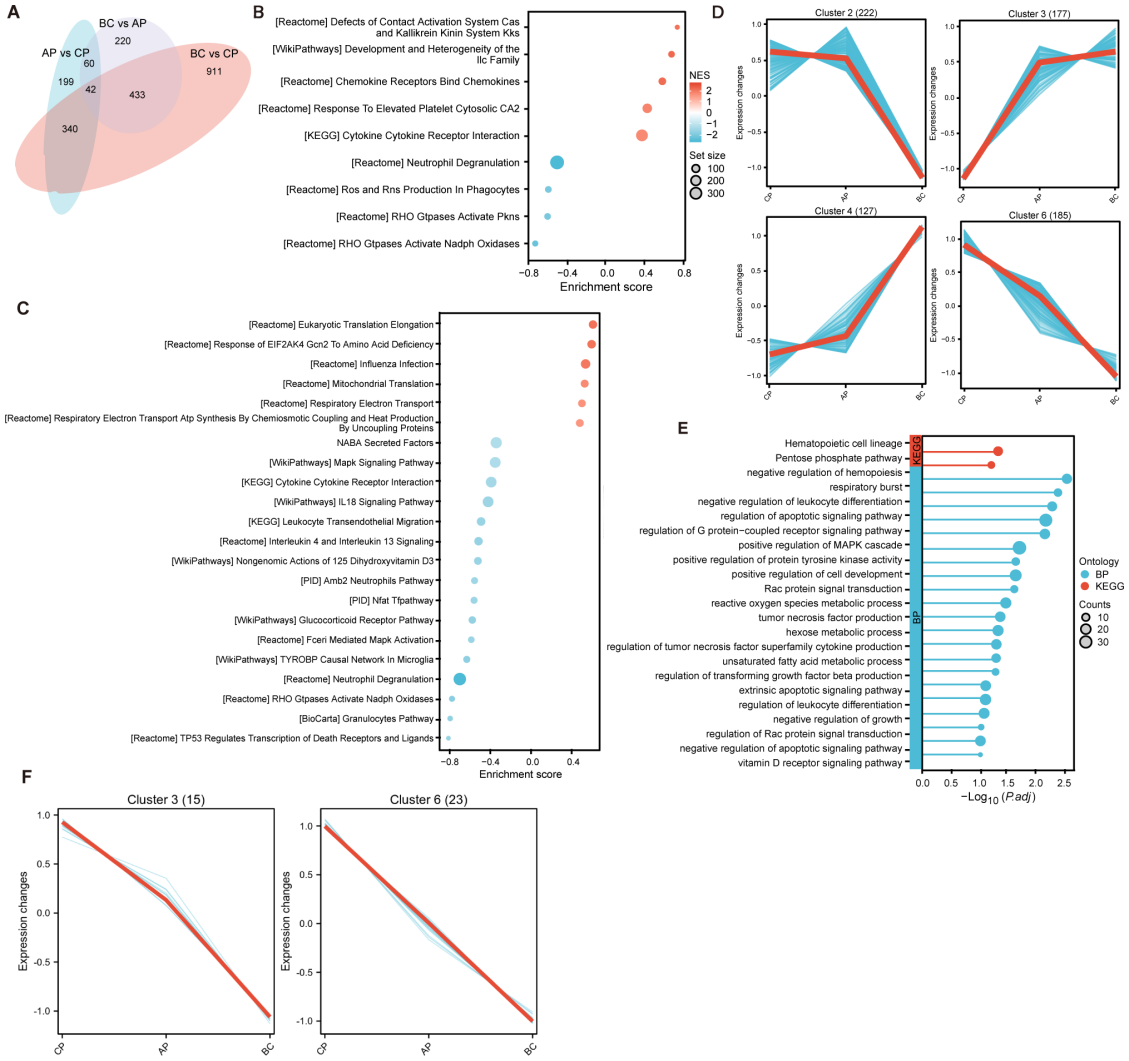
Analysis of differentially expressed genes of CML progression from initial CP to AP and terminal BC. **(A)** Scaled Venn diagram depicting the DEGs identified in each transition. **(B, C)** Gene Set Enrichment Analysis (GSEA) for the transition from **(B)** chronic phase to accelerated phase (CP vs. AP) and **(C)** accelerated phase to blast crisis (AP vs. BC). **(D)** Trend analysis of the 1294 differentially expressed genes (AP vs CP plus BC vs AP, overlaps removed) to illustrate the changes across CP, AP, and BC stages. **(E)** GO and KEGG enrichment analyses of the consistently trending genes in **(D)** illuminate the key pathways driving progression. **(F)** Trend analysis of the 102 differential genes shared between CP vs AP and AP vs BC.

In order to explore the blast-crisis transformation in CML, the GSE4170 dataset from the Gene Expression Omnibus (GEO) database was acquired to screen for key genes. Subsequently, we selected 91 samples out of 121 samples. Differential expression analysis revealed 641 and 755 genes that were differentially expressed in the comparisons of accelerated phase (AP) versus chronic phase (CP) and blast crisis (BC) versus AP, respectively (**Figure 1A** and **Data Sheet 1**). Gene Set Enrichment Analysis (GSEA) was used to annotate the functional pathways associated with these transitions (**Figures 1B, C**). The predominant functional groups comprised nuclear genes expression, mitochondrial metabolism, RNA-binding, adhesion, cytokine-cytokine receptor interaction, and granulocyte pathway, indicating heightened proliferation and metabolism associated with advanced disease. We next conducted a trend analysis of all 1294 differentially expressed genes across the three disease periods, as well as 102 differentially expressed genes within the three-phase intersection (**Figure 1D**). This gene set, characterized by consistent expression trends in the transition from chronic to blast phase, was termed as the “progression-specific” gene set. The gene functions of the progression-specific gene set were categorized based on GO and KEGG functional annotations (**Figure 1E**). In advanced-phase, these primary functional groups encompassed negative regulation of hemopoiesis and differentiation, mitochondrial metabolism, apoptotic signaling pathway, as well as positive regulation of MAPK cascade and protein tyrosine kinase activity. The activation of pathways above-mentioned may enable advancement despite therapeutic inhibition of BCR::ABL activated pathways. In addition, among the 41 intersecting genes from **Figure 1D**, only SOCS2, PTGR2, and CDH2 exhibited elevated expression levels, whereas all remaining genes demonstrated decreased expression in progression (**Figure 1F**). Our analysis indicated that these genes may have played a pivotal role in the advancement of CML.

### 3.2 Elevated expression of N-cadherin in patients with CML blast crisis

**Figure 2:**
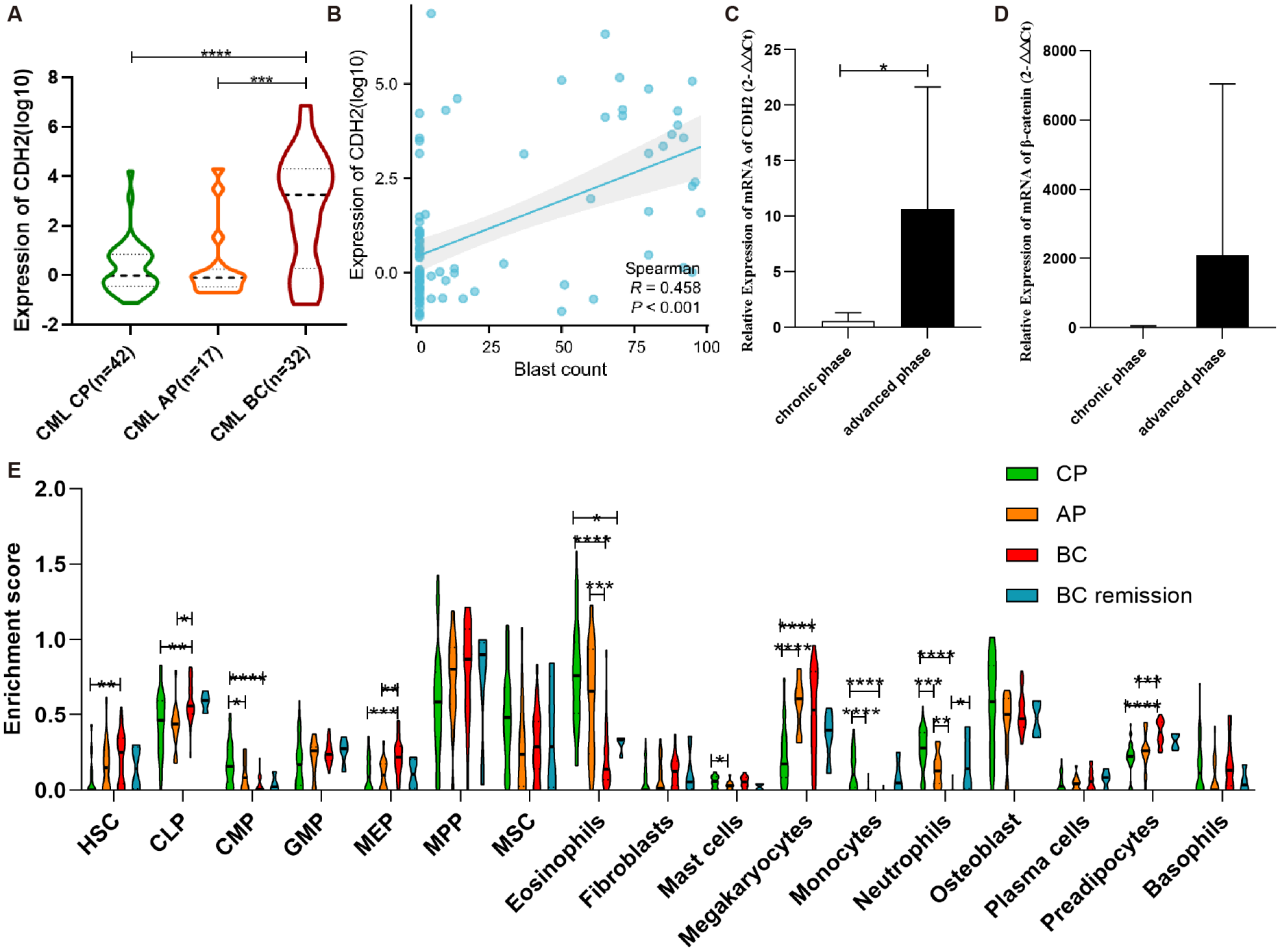
N-cadherin expression and immune infiltration in CML progression. **(A)** Expression trend of N-cadherin across chronic phase (CP), accelerated phase (AP), and blast crisis (BC) from GSE4170. CML-CP (n = 42) vs CML-AP (n = 17) vs CML-BC (n = 32). CP vs BC: *p* < 0.0001, Cohen’s d = 1.41; AP vs BC: *p* = 0.0004, Cohen’s d = 1.14. **(B)** The scatter plot illustrating the correlation between blasts and N-cadherin expression in CML. **(C, D)** Increased expression of **(C)** N-cadherin and **(D)** *β*-catenin in individuals with advanced phase of CML. CML-CP (n = 7) vs CML-AP/BC (n = 7). **(C)**: *p* = 0.0323, Cohen’s d = 1.29; **(D)**: *p* = 0.29, Cohen’s d = 0.60. **(E)** Analysis of immune infiltration in four cohorts of CML. CP (n = 42), AP (n = 17), BC (n = 28), BC -remission (n = 4). For HSC: η^2^_p_ = 0.13; CP vs BC (*p* = 0.0029). For CLP: η^2^_p_ = 0.15; CP vs BC (*p* = 0.0052), AP vs BC (*p* = 0.0015). For CMP: η^2^_p_ = 0.27; CP vs AP (*p* = 0.031), CP vs BC (*p* < 0.0001). For MEP: η^2^_p_ = 0.19; CP vs BC (*p* = 0.0005), AP vs BC (*p* = 0.0049). For Eosinophils: η^2^_p_ = 0.40; CP vs BC (*p* < 0.0001), CP vs. BC remission (*p* = 0.024), AP vs BC (*p* = 0.0003). For Mast cells: η^2^_p_ = 0.11; CP vs AP (*p* = 0.031). For Megakaryocytes: η^2^_p_ = 0.30; CP vs AP (*p* < 0.0001), CP vs. BC remission (*p* < 0.0001). For Monocytes: η^2^_p_ = 0.30; CP vs AP (*p* < 0.0001), CP vs. BC remission (*p* < 0.0001). For Neutrophils: η^2^_p_ = 0.51; CP vs AP (*p* = 0.001), CP vs. BC (*p* < 0.0001), AP vs BC (*p* = 0.0015), BC vs. BC remission (*p* = 0.034). For Preadipocytes: η^2^_p_ = 0.37; CP vs. BC (*p* < 0.0001), AP vs BC (*p* = 0.0006). **(A, C, D)**: Data were analyzed using an unpaired two-tailed Student’s t-test. **(E)**: Data were analyzed using one-way ANOVA followed by Tukey’s *post hoc* test. Data are presented as mean ± SD.*p < 0.05, **p < 0.01, ***p < 0.001, ****p < 0.0001.

Bioinformatic analysis of GSE4170 suggested that CDH2 may act as a key driver in the progression of human CML (**Figure 2A**). Furthermore, correlation analysis indicated a weak relationship between CDH2 mRNA levels and blast percentage (**Figure 2B**). To test this hypothesis, we detected mRNA levels of N-cadherin and *β*-catenin from peripheral blood specimens of CML patients (**Supplementary Table 1**) at different stages of progression (**Figures 2C, D**). qPCR analysis showed that the mRNA level of N-cadherin was low in chronic-phase patients, but significantly higher in patients with advanced phase (**Figure 2C**). In addition, the mRNA level of *β*-catenin was found to be elevated in the advanced phase, although the difference did not reach statistical significance (**Figure 2D**). It was hypothesized that N-cadherin and *β*-catenin may exert a significant influence on the progression to blast crisis of CML. In order to clarify the composition of the immune cells among the bone marrow tissues in different stages of CML progression, we performed an immune infiltration analysis, which was used to assess and quantify the immune cell infiltration, and the immune microenvironment. Enrichment scores for HSC, MEP, and preadipocyte cells were found to consistently correlate with disease progression of CML, demonstrating a significant increasing trend. Conversely, the Enrichment scores of Eosinophils, monocyte, and neutrophil cells exhibited an opposite association with the progression of CML, also showing a statistically significant difference (**Figure 2E**).

### 3.3 Function of N-cadherin in proliferation and differentiation

**Figure 3:**
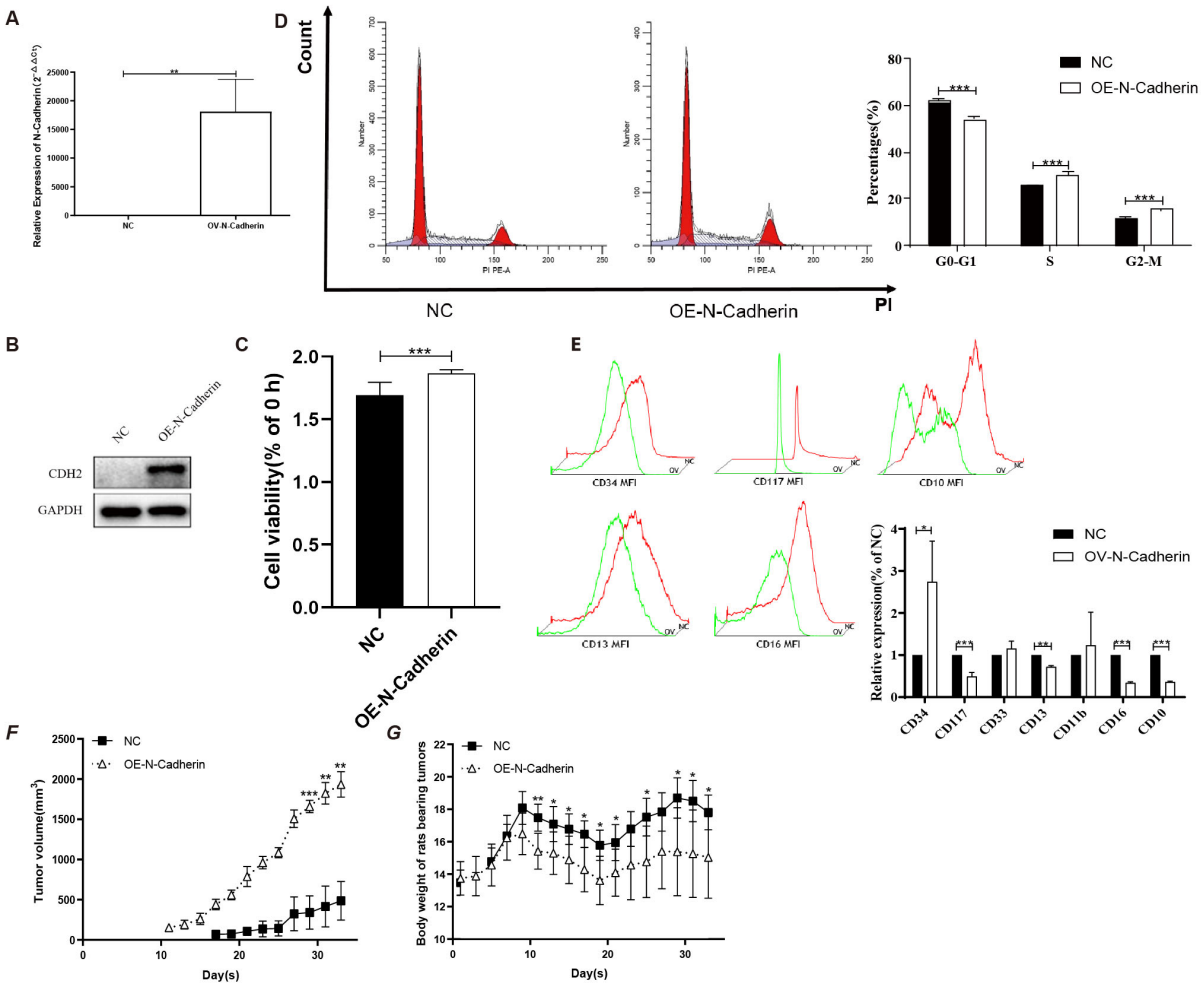
N-cadherin overexpression facilitates proliferation both *in vitro* and *in vivo*. **(A-B)** Validation of N-cadherin overexpression in KU812 cells transfected with the OE-Ncad plasmid. **(A)** Quantitative real-time PCR analysis showing an increase in mRNA levels of N-cadherin in KU812 cells. *p* = 0.005, Cohen’s d = 4.6. **(B)** Western blotting confirming increased N-cadherin protein abundance. **(C)** Cell proliferation assay demonstrated that overexpression of N-cadherin resulted in increased cell proliferation after 48h. *p* < 0.0001, Cohen’s d = 2.5. **(D)** Cell cycle analysis by flow cytometry showed that N-cadherin overexpression promoted cell cycle progression. G0-G1: *p* < 0.0001, Cohen’s d = 8.6; S: *p* = 0.0007, Cohen’s d = 4.7; G2-M: *p* < 0.0001, Cohen’s d = 7.3. **(E)** Analysis of differentiation markers revealed that N-cadherin overexpression inhibited the expression of the myeloid differentiation antigen. CD34: *p* = 0.0328, Cohen’s d = 1.0; CD117: *p* = 0.0003, Cohen’s d = 3.1; CD13: *p* = 0.0027, Cohen’s d = 6.5; CD16: *p* < 0.0001, Cohen’s d = 14.8; CD10: *p* < 0.0001, Cohen’s d = 23.1. **(F)** Subcutaneous tumor growth in nude mice (n=5 per group). N-cadherin overexpression significantly promoted tumor growth. Data from day 29 onwards were analyzed using Two-way repeated-measures ANOVA, which revealed a significant effect of time (η^2^_p_ = 0.37), group (η^2^_p_ = 0.98). **(G)** Body weight of nude mice after inoculation with tumor cells from each group. Two-way repeated-measures ANOVA was used, which revealed a significant effect of interaction (η^2^_p_ = 0.52). Three technical replicates were done. Data are presented as mean ± SD. *p < 0.05, **p < 0.01, ***p < 0.001.

To validate the function of N-cadherin in CML, we constructed an OE-N-cad lentiviral vector and introduced it into KU812 cells harboring at least one Philadelphia chromosome (Ph1). Subsequently, we confirmed that OE-N-cad markedly increased N-cadherin expression by western blot and real-time PCR (**Figures 3A, B**). And then, a CCK8 assay was employed to assess cell proliferation. N-cadherin overexpression promoted cell proliferation after 48h (**Figure 3C**). Due to overexpression of N-cadherin affecting cell division, we further examined the cell cycle analysis by flow cytometry. Compared with control scramble, overexpression of N-cadherin reduced the G1 fraction and increased S/G2/M phases, also indicating enhanced proliferation (**Figure 3D**). Additionally, analysis of cell surface differentiation antigens by flow cytometric analysis revealed that overexpression of N-cadherin impeded cellular differentiation, as evidenced by increased CD34 alongside decreased CD13 and CD10 expression (**Figure 3E**). These findings suggested that cell proliferation and differentiation showed an inverse relationship to a certain extent.

### 3.4 N-cadherin accelerates the formation and proliferation of tumors *in vivo*

In order to further substantiate the impact of cells exhibiting higher expression of N-cadherin *in vivo*, we conducted subcutaneous injections of cells transfected with OE-N-cad lentiviral vector, as well as negative control lentiviral vectors, into nude mice. Tumor monitoring revealed a notably accelerated tumor formation rate in the N-cadherin overexpression group compared to the control group, with 83% (5 out of 6) of nude mice developing visible tumors by day 11, whereas only final 60% in the control group exhibited tumor-bearing mice by day 17 (**Figure 3F**). Moreover, nude mice inoculated with N-cadherin-overexpressing cells displayed faster tumor growth and earlier onset of weight loss compared to those in the control group (**Figure 3G**). These findings strongly support a pivotal role for N-cadherin in promoting KU812 cell proliferation *in vivo*.

### 3.5 MSAB reverses the impact of N-cadherin on cell proliferation and differentiation

**Figure 4:**
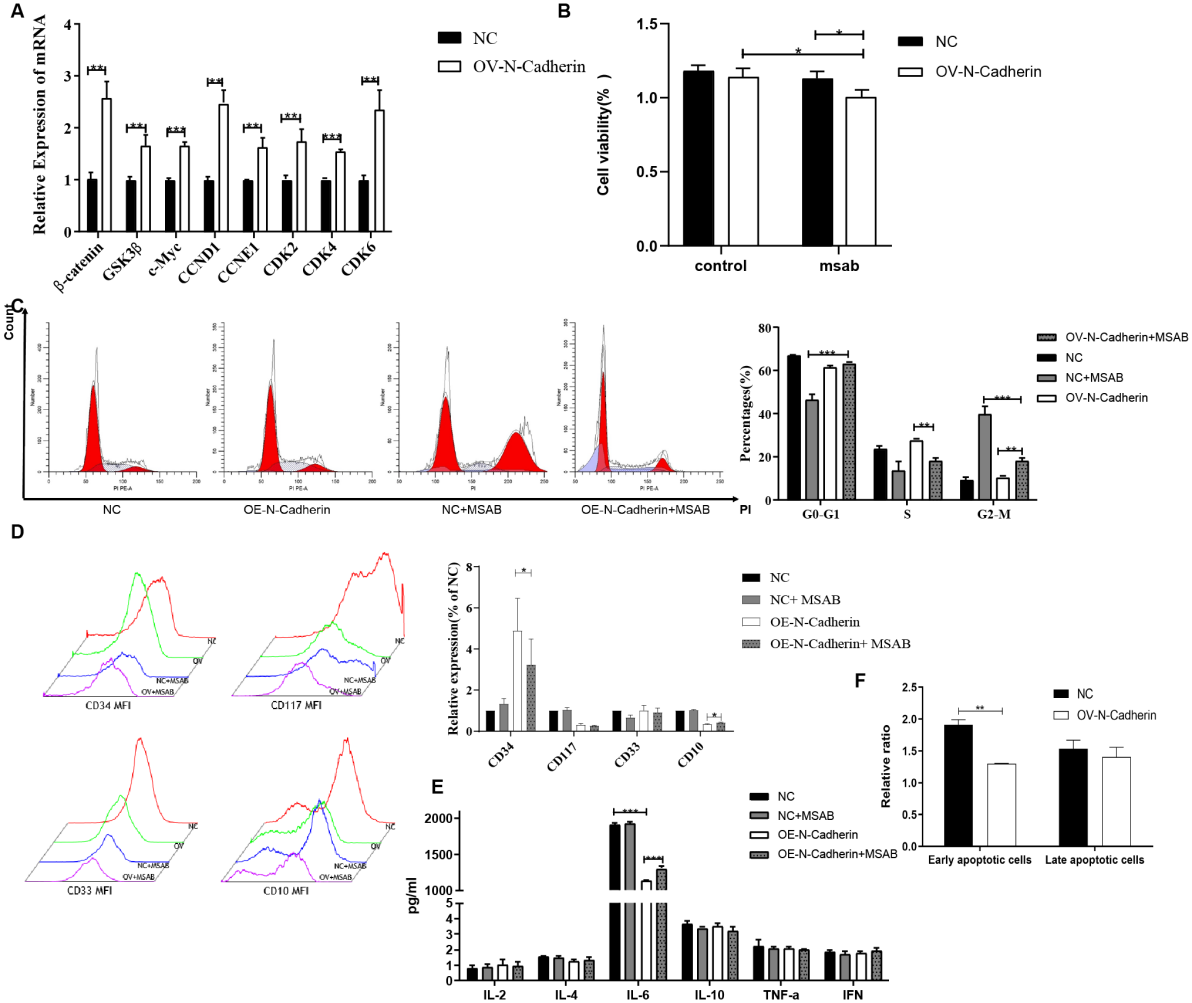
Inhibition of *β*-catenin by MSAB reverses the pro-proliferative and anti-differentiation effects of N-cadherin overexpression. **(A)** N-cadherin overexpression upregulated the mRNA levels of key Wnt/*β*-catenin signaling target genes. Statistical significance was determined using unpaired t-test followed by Benjamini-Hochberg correction for multiple comparisons. **(B)** Cell proliferation assay showed that MSAB suppressed the enhanced proliferation induced by N-cadherin. Data were analyzed by two-way ANOVA followed by Bonferroni’s multiple comparisons test. A significant interaction was found between N-cadherin expression and MSAB treatment (*p* = 0.049, η^2^_p_ = 0.85). OV-N-Cadherin vs OV-N-Cadherin+MSAB: *p* = 0.013, η^2^_p_ = 0.63; NC+MSAB vs OV-N-Cadherin +MSAB: *p* = 0.02, η^2^_p_ = 0.82. **(C)** Cell cycle distribution was analyzed by flow cytometry in KU812 cells following 16-hour treatment with MSAB. Data were analyzed by two-way ANOVA, which revealed a significant interaction between N-cadherin expression and MSAB treatment. G0-G1: η^2^_p_ = 0.96; S: η^2^_p_ = 0.87; G2-M: η^2^_p_ = 0.97. **(D)** Apoptosis assay analyzed by flow cytometry in KU812 cells following 16-hour treatment with MSAB indicated that MSAB promoted cell apoptosis. Data were analyzed using an unpaired two-tailed Student’s t-test. *p* = 0.0016, Cohen’s d = 6.3. **(E)** Flow cytometry analysis demonstrated that MSAB promoted the expression of myeloid differentiation antigens (CD34 and CD10). CD34: *p* = 0.037, η^2^_p_ = 0.25; CD10: *p* = 0.012, η^2^_p_ = 0.13. **(F)** Flow cytometry analysis showed that the inhibition of IL-6 production could be reversed by MSAB. IL-6: η^2^_p_ = 0.94; NC vs OV-N-Cadherin: *p* < 0.0001; OV-N-Cadherin vs OV-N-Cadherin+MSAB: *p* < 0.0001. **(E, F)**. Data were analyzed by two-way ANOVA. Data were representative of three independent experiments and were presented as mean ± SD. *p < 0.05, **p < 0.01, ***p < 0.001.

We observed a significant increase in the transcript levels of *β*-catenin, GSK3*β*, c-Myc, CCND1, CCNE1, CDK2, CDK4 and CDK6 in N-cadherin overexpressing cells as determined by q-PCR (**Figure 4A**). The majority of the genes were found to be downstream targets of the Wnt signaling pathway. Therefore, it was hypothesized that *β*-catenin possibly served as a crucial regulator. To investigate whether β-catenin mediates these effects, we treated cells with MSAB, a specific β-catenin degrader, for 16 hours. CCK-8 and cell cycle assays confirmed that MSAB significantly inhibited cell proliferation, reduced DNA replication, and induced G2/M phase arrest (**Figures 4B, C**). Apoptosis assay by flow cytometry revealed that MSAB treatment significantly induced early-stage apoptosis (**Figure 4D**). Analysis of differentiation markers indicated that MSAB partially restored differentiation, reducing CD34 and slightly elevating CD10 expression (**Figure 4E**). In light of the interplay between cytokines and differentiation, an investigation into cytokines was also conducted by cytokine bead array on flow cytometry. It indicated that N-cadherin overexpression led to a significant decrease in IL-6 levels, while there was a mild increase in IL-6 following MSAB treatment (**Figure 4F**). Collectively, these data demonstrate that *β*-catenin remained is essential for the pro-proliferative and anti-differentiation effects induced by N-cadherin.

### 3.6 N-cadherin plays a role in the progression of CML by modulating *β*-catenin

**Figure 5:**
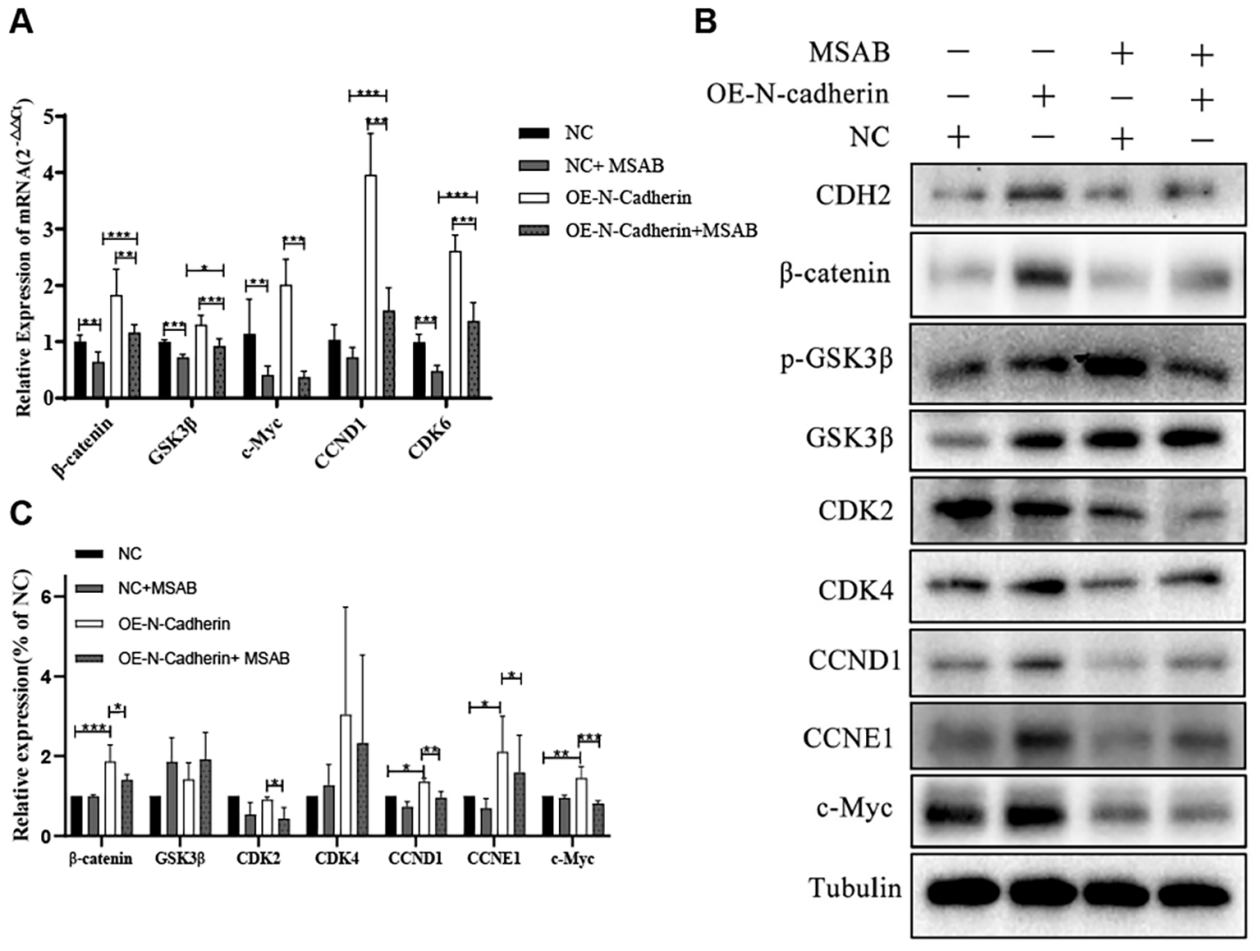
MSAB reverses the N-cadherin-mediated activation of the canonical Wnt/*β*-catenin pathway. **(A)** Quantitative real-time PCR analysis of Wnt pathway target genes in KU812 cells. N-cadherin overexpression significantly upregulated their mRNA levels, which was effectively reversed by MSAB treatment. Data were analyzed by ANOVA followed by Tukey’s multiple comparisons test. *β*-catenin: *p* < 0.0001, η² = 0.75; GSK3*β*: *p* < 0.0001, η² = 0.82; c-Myc: *p* < 0.0001, η² = 0.76; CCND1: *p* < 0.0001, η² = 0.90; CDK6: *p* < 0.0001, η² = 0.93. **(B)** Western blot analysis showed that MSAB downregulated the protein levels of cell cycle regulators (Cyclins) and core components of the canonical Wnt signaling pathway. **(C)** Quantification of the Western blot shown in panel **(B)** Data were analyzed by ANOVA. *β*-catenin: η² = 0.78, *p1* = 0.0007, *p* 2 = 0.05, respectively. CDK2: η² = 0.67, *p* = 0.046. CCND1: η² = 0.87, *p1* = 0.015, *p* 2 = 0.0086, respectively. CCNE1: η² = 0.48, *p1* = 0.049, *p* 2 = 0.043, respectively. c-Myc: η² = 0.76, *p1* = 0.0066, *p* 2 = 0.0004, respectively. Data were representative of three independent experiments and were presented as mean ± SD.*p < 0.05, **p < 0.01, ***p < 0.001.

In this study, we investigated the expression of cyclins and the canonical Wnt/*β*-catenin signaling pathway in hematopoietic malignancy cells. Our qPCR revealed significant up-regulation of cyclins (CCND1, CDK6) and target genes associated with the Wnt/*β*-catenin signaling pathway (such as *β*-catenin and c-Myc) induced by the overexpression of N-cadherin, which were subsequently significantly attenuated following treatment with MSAB (**Figure 5A**). Immunoblotting also indicated that upregulated expression of N-cadherin resulted in elevated protein levels of CCND1, CCNE1, *β*-catenin, and c-Myc. However, the addition of MSAB significantly attenuated the protein levels (**Figures 5B, C**). These results indicate that N-cadherin modulates proliferation and differentiation through the Wnt/β-catenin signaling pathway, suggesting its critical role in CML progression.

## 4 Discussion

Our integrated analysis establishes the N-cadherin/β-catenin axis as a critical driver of chronic myeloid leukemia (CML) blast crisis. Transcriptomic profiling of patient samples revealed consistent upregulation of CDH2 (N-cadherin) throughout disease progression, with peak expression in blast crisis. This clinical observation was functionally validated *in vitro* and *in vivo*: N-cadherin overexpression promoted proliferation, cell cycle entry, and tumor growth while suppressing myeloid differentiation. Crucially, these malignant phenotypes were dependent on β-catenin signaling, as they were effectively reversed by the β-catenin inhibitor MSAB, which restored differentiation and arrested cells in the cell cycle.

Blast crisis is a multi-hit process driven by cooperating mutations (14) (e.g. TP53, ASXL1, RUNX1) and rewired signaling circuits (15) (JAK/STAT, PI3K/AKT, Wnt/β-catenin). Rather than replacing these paradigms, our work positions the CDH2/β-catenin axis as an additional molecular driver, which previously under-appreciated, can operate independently or synergistically with canonical lesions. This view is consistent with recent single-cell studies showing heterogeneous β-catenin activation in BC samples regardless of TP53 status.

Our data align with a growing body of evidence implicating N-cadherin in leukemogenesis. It is highly expressed in various hematological malignancies and is associated with a primitive, CD34+ phenotype in AML (18), consistent with our finding that CDH2 suppresses differentiation antigens (CD13/CD10). Its role extends beyond cell-autonomous effects; CDH2 facilitates bone marrow homing and niche interactions, promoting survival and drug resistance (19, 20). Importantly, the oncogenic function of CDH2 is not limited to the KU812 blast crisis model. Studies in chronic phase (K562) and accelerated phase (LAMA-84) CML cell lines have similarly shown that CDH2 enhances colony formation and proliferation, while its knockdown suppresses growth (21). Furthermore, functional blockade of N-cadherin in human CD34+ progenitors promotes granulocytic differentiation and reduces clonogenicity (22), directly supporting our mechanistic conclusions and underscoring the broader significance of this pathway across CML stages.

We observed that CDH2 overexpression decreases IL-6 secretion and granulocyte differentiation antigens, hinting at a microenvironmental feedback loop. However, we did not perform co-culture assays with marrow stromal cells (e.g. HS-5 or MS-5) or 3-D organotypic systems. Duarte et al. reported that CDH2 blockade in HS-5 co-cultures reduces leukemia-stroma adhesion by 40% and CXCL12 secretion by 38% (20). Future work employing such models is essential to definitively establish how CDH2 signaling influences and is influenced by the microenvironment.

Mechanistically, we provide compelling evidence that β-catenin is the key downstream mediator of oncogenic CDH2 signaling. The upregulation of canonical Wnt targets (CCND1, c-MYC, CDK6) and the profound phenotypic reversal by MSAB firmly support this claim. An intriguing observation was that MSAB-mediated degradation of β-catenin also reduced N-cadherin protein levels, suggesting a potential positive feedback loop wherein β-catenin may transcriptionally or post-transcriptionally stabilize CDH2 (15). Elucidating the molecular basis of this regulation represents a fascinating direction for future research. While genetic knockdown of CDH2 would further strengthen the mechanistic evidence, the significant interaction between N-cadherin overexpression and MSAB treatment observed in our functional assays (**Figures 4**, **5**) strongly suggests that the phenotypic reversal by MSAB is specifically dependent on the N-cadherin/β-catenin axis. This differential effect makes a generalized, off-target mechanism of MSAB unlikely. Furthermore, independent studies using N-cadherin blocking antibodies in human CD34^+^ progenitors have phenocopied the consequences of β-catenin inhibition in our system and corroborating the specificity of this pathway.

While our data demonstrate that enforced CDH2 expression accelerates proliferation and suppresses granulocytic differentiation, two earlier studies reported apparently opposite effects. First, Zhang (23) et al. observed that CDH2 knock-down in K562 chronic-phase CML cells reduced proliferation, implying that CDH2 is necessary for growth (21). Second, work in immature CD34^+^ cord-blood progenitors suggested that CDH2 engagement promotes early erythroid commitment rather than myeloid arrest (22). These discrepancies can be reconciled when the cellular context is considered. Study of Zhang used chronic-phase K562 cells which already express moderate CDH2; removal of this residual CDH2 collapses the limited Wnt/β-catenin tone required for survival. In contrast, we drive CDH2 to supra-physiological levels typical of blast-crisis samples, thereby unleashing excessive β-catenin activation and differentiation block. Puch’s erythroid read-out reflects early lineage priming in normal progenitors, whereas our experimental read-outs focus on myeloid maturation in BCR:: ABL-positive blasts. Importantly, when the same group examined myeloid output in CD34^+^ progenitors, CDH2 blockade indeed promoted granulocytic differentiation (22), aligning with our functional data. Collectively, these comparisons underscore that the oncogenic output of CDH2 is highly context-dependent: moderate expression sustains chronic-phase survival, whereas blast-crisis-level overexpression hijacks the Wnt/β-catenin axis to enforce proliferation at the expense of differentiation.

Of note, the β-catenin inhibitor MSAB only partially reversed the N-cadherin–driven differentiation block, cytokine imbalance and cell-cycle arrest. While MSAB efficiently restored early apoptosis and proliferation, the re-expression of mature myeloid antigens remained modest and IL-6 levels were only marginally rescued. This incomplete phenotypic reversion suggests that additional β-catenin–independent pathways—such as Notch or Jak/Stat signaling—may cooperate with CDH2 to maintain the blast-crisis program, a hypothesis that warrants combinatorial targeting in future studies.

We openly acknowledge the limitations of our study, which also chart a course for future investigation. While our patient data is informative, the cohort size is limited that our findings require further validation in a larger independent cohort. Functional studies were performed predominantly in KU812 cells; although complementary data in K562 and LAMA-84 cells recapitulate the proliferative and clonogenic effects (21), genetic knock-down of CDH2 has not yet been achieved owing to inherently low baseline expression. Our *in vivo* model, while demonstrating the pro-tumorigenic role of CDH2, utilized subcutaneous xenografts. More physiologically relevant systemic or orthotopic models would better recapitulate disseminated disease and microenvironmental interactions. Finally, the broader genomic context of CDH2 overexpression remains unexplored. Integrating transcriptomic or multi-omics profiling in future work could reveal cooperating genetic events.

Despite these limitations, our study unequivocally identifies the CDH2/β-catenin axis as a potent driver of proliferation-differentiation imbalance in CML progression. The efficacy of MSAB in reversing these phenotypes, including in imatinib-resistant models (24), nominates therapeutic targeting of this pathway as a promising strategy for blast crisis. Future efforts should focus on evaluating β-catenin inhibitors, both alone and in combination with TKIs, in primary patient samples and advanced animal models, ultimately aiming to overcome the therapeutic resistance that defines this aggressive phase of CML.

## Data Availability

The original contributions presented in the study are included in the article/**Supplementary Material**. Further inquiries can be directed to the corresponding authors.
