# Supplementary material for: Targeting the N-cadherin/β-catenin axis with MSAB reverses malignant phenotypes in blast crisis of CML
Source: Front Oncol. 2025 Oct 16;15:1657508. doi: 10.3389/fonc.2025.1657508 (PMC12571637; doi:10.3389/fonc.2025.1657508)
Supplement: Supplementary file 1 [file Table1.docx]

Table S1. Clinical metadata of the 14 CML patients enrolled in this study

| Group | Gender | Age(y) | treatment |
| --- | --- | --- | --- |
| CP-01 | Female | 58 | Imatinib-resistant, switched to nilotinib |
| CP-02 | Male | 69 | Hydroxyurea, flumatinib |
| CP-03 | Female | 44 | Imatinib, flumatinib |
| CP-04 | Male | 70 | Nilotinib, flumatinib |
| CP-05 | Male | 37 | Flumatinib |
| CP-06 | Female | 57 | Flumatinib discontinued owing to myelosuppression, then nilotinib |
| CP-07 | Male | 72 | Irregular imatinib initially, then dasatinib |
| AP-01 | Female | 63 | Imatinib mesylate, hydroxyurea |
| AP-02 | Male | 28 | Flumatinib |
| AP-03 | Male | 61 | VP regimen + dasatinib |
| AP-04 | Female | 57 | Flumatinib |
| AP-05 | Male | 61 | Inilotinib, flumatinib |
| BC-01 | Female | 74 | Nilotinib, homoharringtonine, cytarabine |
| BC-02 | Male | 58 | Dasatinib ineffective, tested positive for T315I mutation, switched to hydroxyurea |
