# Supplementary material for: Targeting the N-cadherin/β-catenin axis with MSAB reverses malignant phenotypes in blast crisis of CML
Source: Front Oncol. 2025 Oct 16;15:1657508. doi: 10.3389/fonc.2025.1657508 (PMC12571637; doi:10.3389/fonc.2025.1657508)
Supplement: Supplementary file 2 [file DataSheet1.docx]

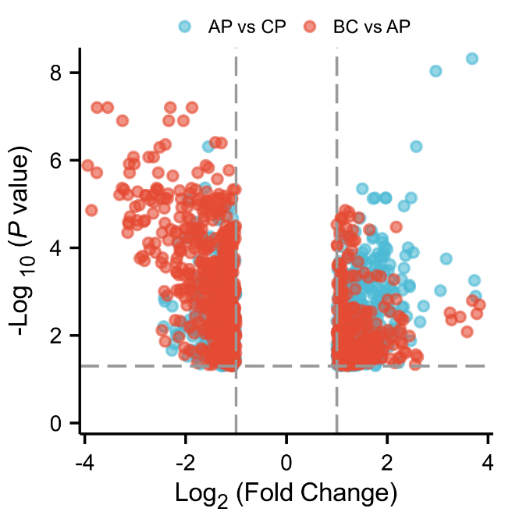


Figure S1. Analysis of differentially expressed genes of CML in progress from initial CP to AP and terminal BC in the GEO database. (S1) Volcano plot of differential gene proﬁles.
